# Metabolic engineering of *Synechocystis* sp. PCC 6803 for the photoproduction of the sesquiterpene valencene

**DOI:** 10.1016/j.mec.2021.e00178

**Published:** 2021-08-13

**Authors:** Maximilian Dietsch, Anna Behle, Philipp Westhoff, Ilka M. Axmann

**Affiliations:** a Institute for Synthetic Microbiology, Department of Biology, Heinrich Heine University Düsseldorf, Düsseldorf, Germany; b Plant Metabolism and Metabolomics Laboratory, Cluster of Excellence on Plant Sciences (CEPLAS), Heinrich Heine University Düsseldorf, D-40001, Düsseldorf, Germany

**Keywords:** Metabolic engineering, Cyanobacteria, Synechocystis, Valencene, Sesquiterpene

## Abstract

Cyanobacteria are extremely adaptable, fast-growing, solar-powered cell factories that, like plants, are able to convert carbon dioxide into sugar and oxygen and thereby produce a large number of important compounds. Due to their unique phototrophy-associated physiological properties, i.e. naturally occurring isoprenoid metabolic pathway, they represent a highly promising platform for terpenoid biosynthesis. Here, we implemented a carefully devised engineering strategy to boost the biosynthesis of commercially attractive plant sequiterpenes, in particular valencene. Sesquiterpenes are a diverse group of bioactive metabolites, mainly produced in higher plants, but with often low concentrations and expensive downstream extraction. In this work we successfully demonstrate a multi-component engineering approach towards the photosynthetic production of valencene in the cyanobacterium *Synechocystis* sp. PCC 6803. First, we improved the flux towards valencene by markerless genomic deletions of *shc* and *sqs*. Secondly, we downregulated the formation of carotenoids, which are essential for viability of the cell, using CRISPRi on *crtE*. Finally, we intended to increase the spatial proximity of the two enzymes, *ispA* and *CnVS*, involved in valencene formation by creating an operon construct, as well as a fusion protein. Combining the most successful strategies resulted in a valencene production of 19 mg/g DCW in *Synechocystis*. In this work, we have devised a useful platform for future engineering steps.

## Highlights

• Engineering strategy to boost isoprene-derived production in cyanobacteria.
• Biosynthesis of commercially attractive plant sequiterpene valencene.
• Markerless genomic deletions of *shc* and *sqs* in *Synechocystis* sp. PCC 6803.
• Downregulation of carotenoid formation using CRISPRi on *crtE*.
• Heterologous *ispA* and CnVS operon and fusion protein.

## 1 Introduction

Cyanobacteria are known for their unique ability of oxygenic photosynthesis among bacteria. Thus, they are becoming increasingly important in biotechnological applications and for generating sustainable energy. Unlike plants, cyanobacteria can be cultivated in huge salt water basins, even in desert regions, solely with sunlight and CO_2_ from the air or from connected power plants and, thus, do not compete with agricultural land and food production Furthermore, extraction of plant secondary metabolites has proven to be inefficient, as it has yielded only small amounts of the desired products thus far. Here, cyanobacteria represent excellent candidates for the expression of plant biosynthetic genes and gene clusters due to their ancestral relationship to plant chloroplasts. In recent years, continuous efforts have been put into developing industrially viable strains of cyanobacteria for the sustainable production of various fine chemicals, secondary metabolites, and other compounds (Jodlbauer et al., 2021; Liu et al., 2021). Advances in synthetic microbiology and increasing availability of new genetic tools for this important group of organisms enable even more innovative solutions.

In terms of structural diversity, terpenoids comprise an extremely versatile class of compounds. Naturally, the terpenoid backbones in cyanobacteria are generated via the methyl-erythritol-phosphate (MEP-) pathway, which produces the central terpenoid precursors IPP and DMAPP. By subsequent addition of another precursor, GPP (C10), the precursor for monoterpenes, FPP (C15) the precursor for sesquiterpenes and triterpenes, such as hopanoids, and GGPP (C20), the precursor for di- and tetraterpenes, to which the carotenoids belong, are generated. One prominent example for natural sesquiterpene production is geosmin found in several Cyanobacteria species, which is responsible for the characteristic earthy smell in water bodies (Lee et al., 2017). Sesquiterpenes are especially convenient for the heterologous production in microorganisms because they are often volatile, eliminating the necessity for costly extraction methods and downstream processing. Naturally, they are often found in plants, where they may function as defensive agents against predators. In industry, sesquiterpenes are used as flavor and fragrance additives and have been successfully produced in numerous microbial hosts, with very different yields.

The first metabolic engineering efforts for the production of sesquiterpenoids were made in *Escherichia coli (E. coli)*, where amorphadiene, the precursor of the antimalarial drug artemisinin, was produced via heterologous expression of the complete mevalonate pathway from the yeast *Saccharomyces cerevisiae (S. cerevisiae)* (Newman et al., 2006). Using a combination of metabolic engineering and a two-phase cultivation system, a total yield of ~0.5 g/L product was achieved. This product titer was even further improved by the introduction of metabolically more active enzymes, as well as an improved growth media composition (Tsuruta et al., 2009).

*S. cerevisiae*, as well as other fungal species, has also successfully been applied for the production of sesquiterpenes such as valencene. Similar to previous efforts, enhancing the flux through the native isoprenoid biosynthesis pathway by overexpressing each gene had an advantageous effect on valencene product yield. In addition, by repressing essential genes that normally diverted some of the FPP precursor away from the desired product, valencene yield increased even more. The highest product yield in yeast, ~540 mg/L valencene, was again achieved by a combination of genetic engineering and optimization of media composition and cultivation (Chen et al., 2019). Recently, the corn smut fungus *Ustilago maydis* was explored as a microbial production host for sesquiterpenes, due to previous successes in biotechnological applications using this host (Lee et al., 2020).

Another strategy to increase the utilization of precursors towards desired products instead of native off-target pathways is to increase the spatial proximity of two sequential enzymes. In yeast, this was achieved by fusing FPP-synthase, encoded by *erg20*, with the heterologous sesquiterpene synthase, producing germacrene A in one case, and patchoulol in another (Albertsen et al., 2011; Chen et al., 2019).

This method of creating a chimeric enzyme showed success, with all fusion variants leading to an overall increase in product yield. In another study, heterologous production of carotenoids was achieved through bi- and tridomain fusion proteins, further demonstrating the importance of spatial proximity in metabolic pathways (Rabeharindranto et al., 2019).

Photoautotrophic bacteria, cyanobacteria in particular, have shown promising results in terms of production (Angermayr et al., 2015). Since they are natural terpene producers, they are excellent candidate chassis for metabolic engineering. In terms of product yield, they were able to compete with heterologous hosts under standard laboratory conditions. For example, the two sesquiterpenoids bisabolene and patchoulol were produced under high density conditions, yielding ~179.4 mg/L and 17.3 mg/L, respectively (Dienst et al., 2020). Another study showed successful production of various triterpenes from one key precursor in *Rhodobacter capsulatus* and *Synechocystis* sp. PCC 6803 (*Synechocystis* hereafter), indicating efficient exploitation of the native terpene pathway of photosynthetic organisms through genetic engineering (Loeschcke et al., 2017). Here, we present a multi-component approach towards the photosynthetic production of valencene. First, we applied metabolic engineering to generate a strain with a more favorable flux towards the precursor FPP by markerless genomic deletions. Secondly, we used CRISPRi to downregulate the formation of carotenoids, which are essential for viability of the cell. Finally, we applied two strategies to increase the spatial proximity of the two enzymes involved in valencene formation by creating an operon construct, as well as a fusion protein to increase the flux from FPP to the final precursor, valencene. This work successfully demonstrates heterologous production of the sesquiterpene valencene in the cyanobacterium *Synechocystis* using different engineering approaches.

## 2 Material & methods

### 2.1 Plasmid and strain construction

A detailed list of all relevant genetic modules and information regarding their origin, is provided in the Supporting Information (Table S2).

The previously published pSHDY-Prha-mVenus_rhaS (Behle et al., 2020) (Addgene #137662) was slightly modified by excising the spectinomycin resistance cassette and replacing it with a nourseothricin resistance cassette, thereby creating an alternative plasmid we termed pSNDY.

Synthetic, codon-optimized genes were synthesized by IDT. Relevant genetic components were amplified and fused using overlap extension PCR when necessary, (dx.doi.org/10.17504/protocols.io.psndnde).

and integrated into the pSNDY backbone, either via Gibson assembly (dx. doi.org/10.17504/protocols.io.n9xdh7n), or using restriction/ligation cloning.

Plasmids were transferred to *Synechocystis* sp. PCC 6803 wild-type using triparental mating (dx.doi.org/10.17504/protocols.io.psndnde).

pMD19T-psba1-Ppsba2-dCas9-SpR was a gift from Paul Hudson (Addgene plasmid # 73220; http://n2t.net/addgene:73220; RRID:Addgene_73220).

### 2.2 Culture conditions

For pre-culturing and growth experiments, *Synechocystis* was cultivated in BG11 medium (Stanier et al., 1979). Standard cultivation was performed at 30 °C with 150 rpm shaking and continuous illumination of ~80 μE m–2 s–1. Aeration was ensured by continuous shaking and CO_2_ enriched air (0.5%). Whenever necessary, appropriate antibiotics were added to the different strains. Pre-culturing was performed in 100 ml baffle-free Erlenmeyer shaking flasks with 20 ml cell suspension for three days. After adjusting all different strains on the OD growth experiments were performed after one additional day of pre-culturing. For this, 4 ml cultures were incubated in 6-well plates for 48 h with a start OD_750_ of 0.5 in biological triplicates. To avoid loss of the volatile product valencene, cultures were overlaid with 20 % dodecane.

### 2.3 Biomass measurements (DCW, OD, spectra)

Optical density and whole cell spectra measurements were performed in the SpEcoRd 200 plus and diluted if necessary. To determine the cell dry weight (CDW) 2–3.5 ml cell culture was pelleted for 3 min at maximum speed. After washing the pellet with PBS buffer, the pellet was resuspended in ~50 μl water and transferred to a pre-weighed PCR tube, where it was dried at 60° overnight prior to weighing.

### 2.4 Microscopy

Cells were analyzed phenotypically using the bright field setting of a Zeiss AxioScope.A1, under 400-fold magnification.

### 2.5 Pigment quantification

0.2–0.5 ml of each culture was sampled after 48 h at the end of the growth experiment. The sample was centrifuged for 5 min at 14,000 g and 4 °C. The supernatant was discarded and the pellet resuspended in 100 μl water. The samples were frozen at −20 °C until further processing. 900 μl of 100% methanol was added and the sample was mixed by vortexing. After incubation with gentle shaking for 30 min at 4 °C, the sample was centrifuged at 14,000 g for 5 min. The supernatant was transferred to a cuvette and the absorbance spectrum was measured from 400 nm to 750 nm. The absorbance spectra were divided by the OD_750_ or CDW and the amount of chlorophyll *a* in the sample was quantified by the absorbance maximum of chlorophyll *a* at 665 nm (A_665nm_) using following equation (Lichtenthaler and Buschmann, 2001):

### 2.6 Chlorophyll content [μg/ml] = 12.66 μg/ml ∗ A_665 nm_

The amount of carotenoids in the sample was quantified by the absorbance maximum of the sum of carotenoids at 470 nm (A_470nm_) and a correction term considering absorbance of chlorophyll *a* at 470 nm (c(Chl a): concentration of chlorophyll *a* in the sample) using the following equation:

Carotenoid content [mg/ml] = (1000 μg/ml ∗ A_470 nm_ −1.91 ∗ c(Chl))/225.

### 2.7 RNA extraction & qRT-PCR

RNA extraction was performed according to (Pinto et al., 2009). Briefly, 0.2–1 ml cell culture was collected and pelleted for 3 min at maximum speed at 4 °C. After discarding the supernatant, the pellet was resuspended with 0.5 ml PGTX and incubated at 95 °C for 5 min. After cooling on ice, 350 μl chloroform/isoamyl alcohol were added and the mixture was incubated shaking gently at room temperature for 10 min. To separate the aqueous from organic phases the mixture was centrifuged for 10 min at maximal speed at 4 °C. The upper phase was transferred to a fresh tube and 1 vol chloroform/isoamyl alcohol added. After repeating the centrifugation step the upper phase was again transferred and precipitated with 3 vol of 100 % ethanol sodium acetate at −20 °C overnight. The RNA was pelleted for 30 min at maximum speed and 4 °C, washed twice with 70% ethanol and resuspended in RNase-free water.

RNA was DNaseI-digested using commercial DNaseI from ThermoFisher (EN0525), according to the manufacturer's specifications. DNaseI-digested RNA was phenol/chloroform extracted again to remove the DNaseI.

For cDNA synthesis, the commercial RevertAid RT from ThermoFisher (K1621) was used according to the manufacturer's specifications.

qRT-PCR was performed using the DyNAmo ColorFlash SYBR™ Green qPCR-Kit (ThermoFisher, F416L), according to the manufacturer's specifications.

### 2.8 GC-MS for the quantification of volatile sesquiterpenoids

100 μL dodecane overlay fractions were collected in micro inserts inside 1.5 mL clear glass GC vials. 2 μL of the sample were diluted 1:50 in HPLC grade hexane (Th. Geyer GmbH, Germany) prior to injection. 1 μl of the diluted was injected with an MPS autosampler with automatic liner exchange system in conjunction with a cold injection system (Gerstel) in splitless mode (ramping from 50 °C to 250 °C at 12 °C s^−1^) into the GC with a helium flow of 1 ml min^−1^. Chromatography was performed using a 7890B GC system (Agilent Technologies) with a HP-5MS column with (5%-phenyl)-methylpolysiloxane film (Agilent, 19091S-433, 30 m length, 0.25 mm internal diameter, 0.25 μM film). The oven temperature was held constant at 70 °C for 2 min and then ramped at 12.5 °C min^−1^ to 320 °C at which it was held constant for 5 min; resulting in a total run time of 27 min. Metabolites were ionized with an electron impact source at −70 eV and 200 °C source temperature and recorded in a mass range of *m/z* 60 to *m/z* 800 at 20 scans per second with a 7200 GC-QTOF (Agilent Technologies) after a solvent delay time of 8 min. Compound identification was conducted via MassHunter Qualitative (v b08.00, Agilent Technologies) by comparison of mass spectra to the NIST14 Mass Spectral Library (https://www.nist.gov/srd/nist-standard-reference-database-1a-v14) and validated by retention time comparison with chemical reference substances (Sigma-Aldrich, #06808). Peaks were integrated using MassHunter Quantitative (v b08.00, Agilent Technologies). The concentration was determined via external calibration. The calibration curve was generated with 8 points from 0.1 μM to 20 μM with a quadratic curve fit and 1/x curve fit weight. After direct measurement of valencene in the dodecane layer, molar concentrations were calculated to mg valencene/L *Synechocystis* culture. To determine whether valencene was lost over time via evaporation or degradation, dodecane with 225 μM valence was measured directly and compared to a dodecane layered cell culture with 225 μM valence and cultivated for 48 h. Technical triplicates were cultured and measured, and there was no significant difference detected between the samples (Fig. S6).

### 2.9 Total protein isolation and Western Blot analysis

Total protein was extracted from cultured and induced *Synechocystis* cultures as described (dx.doi.org/10.17504/protocols.io.ps6dnhe). Protein concentration was determined according to Lowry et al. using a BSA standard. 20 μg total protein was loaded on an SDS gel, transferred to a PVDF membrane, UV-crosslinked, and the presence of the IspA:CnVS fusion protein, as well as IspA only from the operon construct, was detected using a monoclonal anti-FLAG-M2-alkaline-phosphatase antibody (Sigma, A9469) as primary, and an anti-mouse antibody as the secondary antibody.

## 3 Results & discussion

**Fig. 1:**
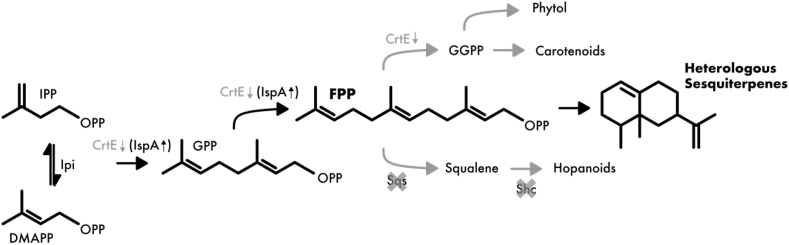
Isoprene pathway from *Synechocystis* with optimizations done in this work. Abbreviations used: IPP = isopentenyl diphosphate; DMAPP = dimethylallyl diphosphate; GPP = geranyl diphosphate; FPP = farnesyl diphosphate; Ipi = isopentenyl diphosphate delta isomerase; CrtE = geranylgeranyl pyrophosphate synthase; Sqs = squalene synthase; Shc = squalene hopene cyclase; IspA = farnesyl diphosphate synthase. Crossed out target = gene deletion. Down arrow = repression target. Upward arrow = overexpression/rescue.

The central terpenoid pathway in *Synechocystis* starts with IPP and DMAPP, which are derived from the MEP-pathway. A single gene, *crtE*, is responsible for the elongation of terpene precursors towards GPP, FPP, and GGPP (Fig. 1). Next to GGPP, another downstream metabolic product of FPP is squalene, which is converted to hopanoids.

In the following, we demonstrate various strategies to divert metabolic flux towards heterologous sesquiterpenes (Fig. 1, black components), effectively eliminating undesired side products (Fig. 1, gray components).

### 3.1 Modulating the internal precursor pool by genomic gene deletion of squalene synthase and squalene hopane cyclase

**Fig. 2:**
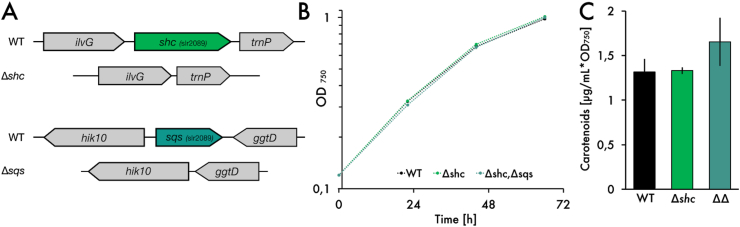
Knockout strategy and growth/pigment analysis of mutants. A: Schematic overview of markerless mutant genotypes. B: Comparison of growth between wild type and mutants. C: Carotenoid content of the three different strains. Results represent the mean of three biological replicates. WT = wild type, Δshc = squalene-hopene-cyclase deletion, ΔΔ = squalene-hopene-cyclase and squalene synthase deletion.

In order to divert metabolic flux away from undesired side products and towards farnesyl pyrophosphate (FPP), which is the central precursor for sesquiterpenes (Fig. 1), we applied two strategies. First, we performed markerless deletions of two genes, squalene synthase (*sll0513*, *sqs*), which is responsible for the conversion of FPP to the triterpene squalene, and the gene directly downstream, squalene hopane cyclase (*slr2089*, *shc*), which further converts squalene to hopanoids. A *shc* knock-out was previously performed in order to accumulate squalene, and a 70-fold increase was demonstrated using this deletion mutant (Englund et al., 2014). We hypothesize that an additional *sqs* deletion might lead to an accumulation in FPP in a similar manner. We further improved the initial strain design by performing markerless gene deletions, which are of special interest because resistance cassettes can be recycled, instead of being occupied indefinitely within the genome. Thereby, multiple different alterations in one strain are possible. Each markerless deletion was performed in two sequential steps as previously described (Viola et al., 2014); first, a CmR-sacB cassette, flanked by the neighboring genomic regions and including a partially overlapping fragment thereof, was introduced into wild type *Synechocystis*. By gradually selecting on higher chloramphenicol concentrations, complete genome segregation was achieved. In a second step, counter-selection of segregated clones on solid media containing sucrose, but no chloramphenicol, was carried out, thereby eliminating cells still carrying the CmR-sacB cassette and selecting for a second double-crossover event between the partial overlaps. The schematic genotype of the double mutant is shown in Fig. 2A.

To ensure complete deletion of both genes, the lack of transcripts was verified via qRT-PCR (Table S1) correctly segregated mutants were verified via colony PCR (Fig. S1A).

The fully segregated strain was then compared to the wild type strain in terms of growth and pigment composition. While there was no discernible difference in growth (Fig. 2B), the double mutant showed a visible shift in carotenoids (Fig. S1 C, D), suggesting an increase in metabolic flux towards the central carotenoid precursor, GGPP, which is derived from FPP (Fig. 1). Upon further investigation via pigment extraction, this observation was confirmed; the double mutant showed an increase in carotenoid content (Fig. 2C). Due to the previously described toxicity of FPP in *E. coli* (Dahl et al., 2013)*,* an enrichment of this intermediate seems implausible and a further conversion to harmless carotenoids seems to be likely.

**Fig. 3:**
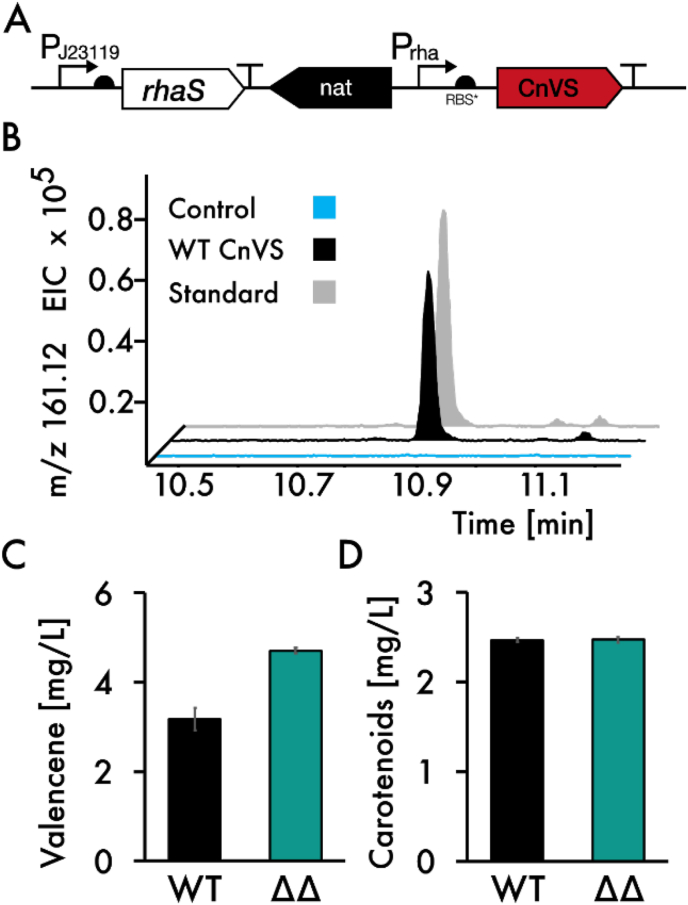
Comparison of productivity between wild type and mutant. A: Construct overview. B: Detection of valencene by GC-MS analysis. Dodecane layer of the engineered Synechocystis strain (WT CnvS) after 48 h cultivation with 5 μM rhamnose induction, compared with a standard (225 μM) and the dodecane layer of the cultivated wild type strain (Extracted ion chromatogram, m/z 161.12). C: Valencene production in wild type (WT) and the Δshc/Δsqs mutant strain (ΔΔ). D: Carotenoid content in wild type (WT) and the Δshc/Δsqs mutant strain (ΔΔ). Results represent the mean of three biological replicates.

To test whether this increased carotenoid content could be translated to an increased FPP availability for sesquiterpene production, the wild type and mutant expressing valencene synthase (CnVS) from *Callitropsis nootkatensis* (Beekwilder et al., 2014) under the rhamnose-inducible promoter (Behle et al., 2020) (Fig. 3A) were cultured alongside in 6-well plates for two days. To avoid loss of the volatile product valencene, cultures were overlaid with 20 % dodecane. The dodecane layer was then sampled and quantified directly using GC-MS.

The identification of valencene in all strains was performed by comparing retention time and mass spectra with those of a commercial standard (Fig. S4). In Fig. 3B, the WT expressing *CnVS* as well as a negative control is shown in the extracted ion chromatogram (m/z 161.12) as an example.

Remarkably, the double mutant showed a ~40% increase in valencene production compared to the wild type (Fig. 3C). In contrast to before (Fig. 2C), the mutant now expressing *CnVS* did not show an increase in carotenoid content (Fig. 3D). In all likelihood, the excess precursor pool that was diverted towards carotenoid production before was now successfully used by CnVS. Due to the promising production increase in the mutant, we exclusively used the double knockout mutant background for the following experiments.

### 3.2 Enhancing the FPP precursor pool by conditional repression of the essential gene *crtE*

To further exploit the available carotenoid pool, we aimed at reducing the conversion of FPP to GGPP. In *Synechocystis*, a single gene, *crtE*, is responsible for the consecutive condensation of IPP and DMAPP to GPP, FPP and finally to GGPP (Lin et al., 2017), the precursor for diterpenoids, including the chlorophyll phytol tails, and tetraterpenoids, such as carotenoids (Fig. 1A). In contrast, genes from heterotrophic species, such as *ispA* from *E. coli*, only perform these conversions up until FPP (Reiling et al., 2004). Since GGPP-derived pigments are essential for cyanobacterial viability, we decided to reduce *crtE* expression via inducible, dCas9-based CRISPRi, and then introduce a heterologous FPP-synthase to increase the relative amount of FPP compared to GGPP.

**Fig. 4:**
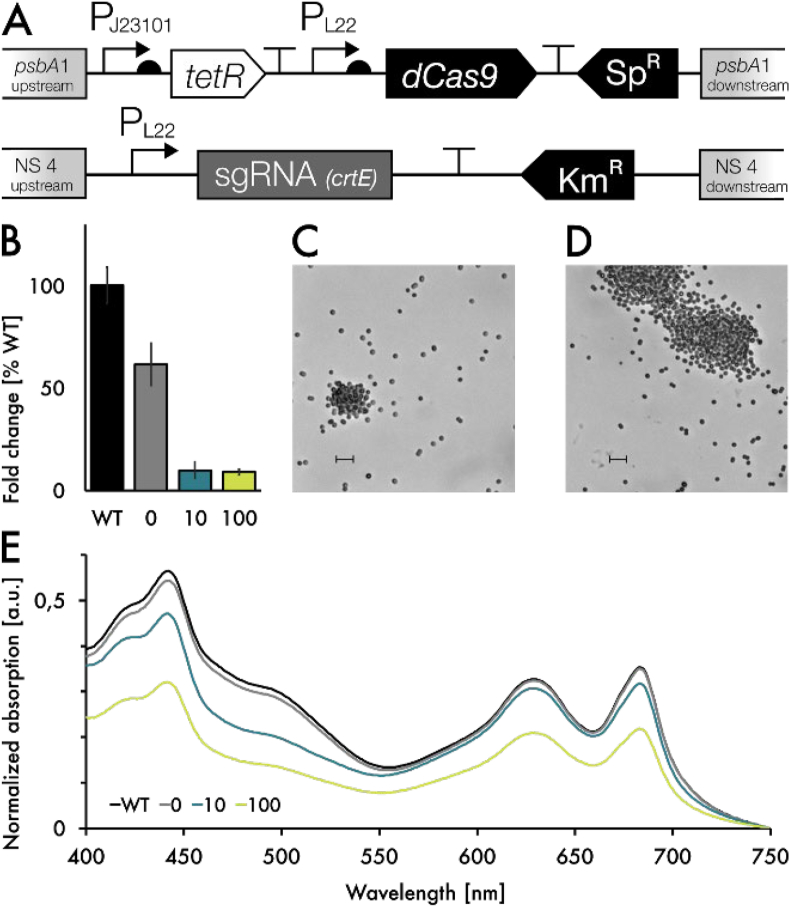
CrtE gene repression in Synechocystis. A: Construct overview. B: CRISPRi knockdown of Geranylgeranyl pyrophosphate synthase (CrtE) using the PL22 promoter with 0, 10 and 100 ng/ml anhydrotetracycline (aTc). Transcripts measured by RT-qPCR after 24h of cultivation compared to the induced (100 ng/ml) control strain denoted as WT (containing only dCas9, but no sgRNA). Results represent the mean and standard deviation of three biological replicates and three technical replicates each. C/D: Bright field microscopy picture after 24 h cultivation of the strain with 10 ng/ml (C) or 100 ng/ml (D) aTc induction. Magnification ×400, scale bar 10 μm. E: Whole cell absorption spectra analysis. Cultures were adjusted for OD750 prior for measurement and values were baseline corrected. CrtE reduction leads to a blueish culture color. (For interpretation of the references to color in this figure legend, the reader is referred to the Web version of this article.)

We chose an sgRNA from a previously published work to target *crtE* (Yao et al., 2020), as well as the aTc-inducible dCas9 system from Yao et al. (2016) (Fig. 4A).

Interestingly, the qRT-PCR with *crtE*-specific oligonucleotides shows a repression down to <10 % of the wild type level at much lower inducer concentrations of 10 ng/mL aTc, despite a reported >90 % repression at concentrations as low as 100 ng/mL aTc. Notably, the uninduced *crtE*-repression strain already shows a 40 % reduction of gene expression compared to the wild type (Fig. 4B). Consistent with published results, induction with 100 ng/mL aTc shows almost complete repression of *crtE*. While the pigment composition of the uninduced strain resembles the wild type, an aTc-dependent effect on both chlorophyll and carotenoids can be observed (Fig. 2C). General pigmentation is severely affected at 100 ng/mL aTc, whereas only carotenoids are affected at 10 ng/mL aTc. This was further confirmed via pigment extraction (Fig. S2 A). In addition, a severe photoprotective phenotype, where the cells form aggregates, was observed at 100 ng/mL (Fig. 4D). This also occurred at 10 ng/mL aTc, but much less frequent and with smaller clumps (Fig. 4C). Interestingly, when culturing the strains in 6-well plates, OD_750_ was almost not affected at all (Fig. S2 B).

It is possible that the slight phenotype observed at 10 ng/mL aTc might become more severe over longer periods of time, since the expression levels appear to be saturated at the lowest concentration used. Likely, this is due to phenotypical changes taking longer than changes in expression. Another possibility is faster repression by higher aTc concentrations, resulting in a higher initial amount of CrtE protein at 10 ng/mL aTc compared with 100 ng/mL, which would take longer to be diluted out via cell division. On the other hand, since aTc is light-sensitive, the effect is likely transient and cells may recover from both their phenotype and their carotenoid deficit. Since industrial applications rely on robust strains, ideally without the necessity of adding costly inducer compounds, further fine-tuning might be of interest to achieve a constitutively downregulated *crtE* gene, while still maintaining cell viability and productivity. Since the uninduced control already shows a noticeable decrease in expression, using a stronger promoter for the control of the CRISPRi system might already be sufficient.

Nonetheless, we were able to demonstrate that tuned down-regulation of *crtE* leads to a reduction of carotenoids, while maintaining almost wild type levels of chlorophyll, as well as a wild type-like performance in terms of cell growth, and that by applying this strategy, we likely were able to enhance precursor availability for heterologous biosynthetic pathways upon introduction of alternative prenyltransferases.

### 3.3 Exploiting the carotenoid pool for the production of valencene

Since the newly engineered *crtE* knock-down strain lacks GGPP, but also the desired FPP precursor, we introduced the heterologous *ispA* gene from *E. coli*, which is functionally homologous to *crtE*, but unable to produce GGPP (Reiling et al., 2004).

**Fig. 5:**
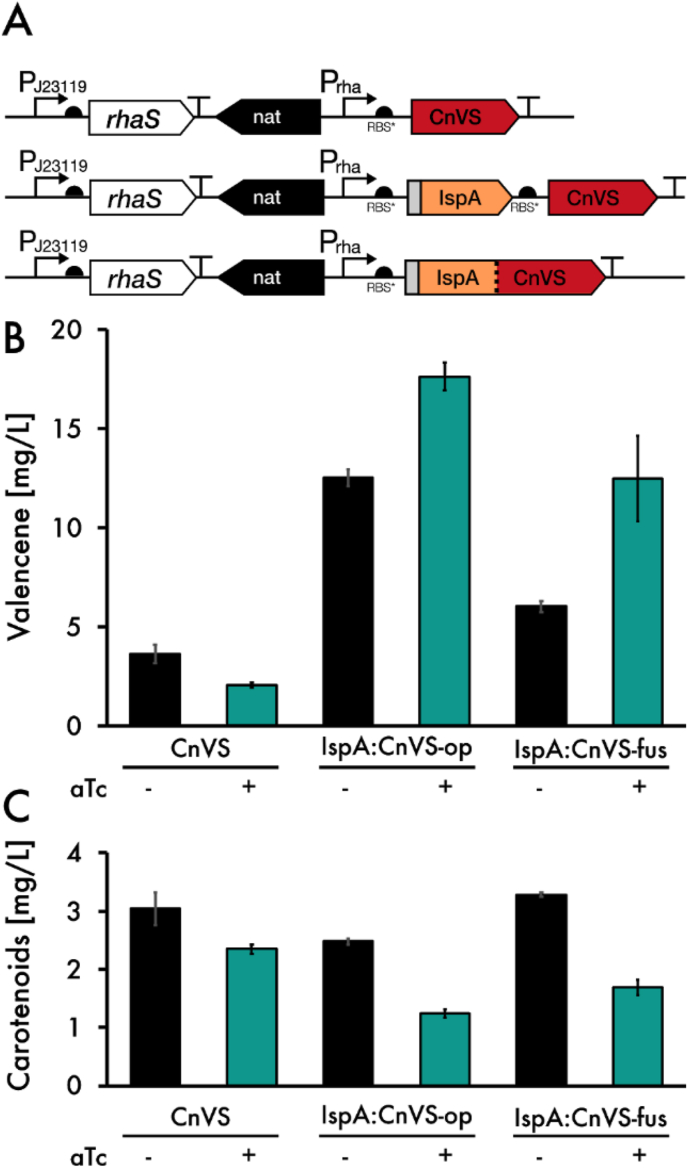
Performance of the crtE repressed valence production. A: Construct overview. B: Valencene production in the CnVS, the CnVS-ispA-operon and CnVS-ispA-fusion strain. All strains are in the ΔΔ and dCas9/CrtE sgRNA background. + indicates the induction with 10 ng/ml aTc. Additionally, all strains were induced with 5 mM rhamnose. C: Carotenoid level of strains described. Results represent the mean and standard deviation of three biological replicates.

To favor conversion of IPP and DMAPP towards valencene, we applied two strategies. First, we generated an ispA-CnVS protein fusion construct with a GGGGS linker in between to strongly increase proximity between the two enzymes the linker was chosen because it showed the most promising results in previous works (Hu et al., 2017). Second, we cloned the same genes in an operon (Fig. 5A). In the operon case, the enzymes were able to retain their full functions, while still being translated from the same mRNA, thereby optimizing spatial and temporal proximity to each other without potential compromise of function. The constructs were designated IspA:CnVS-fus and IspA:CnVS-op, respectively. In all variants, heterologous genes were controlled by the strong inducible promoter, P_rha_. Fig. 5A outlines the construct design. To verify the production of soluble protein, we included an N-terminal FLAG-tag upstream of *ispA*. Western Blot analysis confirmed the presence of both the chimeric protein in ispA:CnVS-fus, as well as *ispA* in ispA:CnVS-op (Fig. S3 A). We also included a control with only CnVS to quantify the performance of the enzyme on its own in each background strain.

Cultures were grown as described earlier, and dodecane fractions were sampled after 48 h, before quantifying OD_750_ and density-adjusted spectra, as well as sampling for pigment extraction, quantification of dry cell weight (DCW).

As hypothesized, *crtE*-knockdown strains expressing only *CnVS* performed poorly in terms of valencene production. Induction of *crtE* repression via aTc led to a decrease in both valencene yield and carotenoids. This was expected, since all intermediates within the terpenoid pathway should be affected by a repression of *crtE*.

Coexpression of *ispA* and *CnVS*, both as an operon and a fusion protein, resulted in an increased amount of valencene. Especially in IspA:CnVS-op, production increased by about 3.5-fold compared to the strain expressing only *CnVS*. The increase in IspA:CnVS-fus was less apparent with a 1.7-fold change in valencene.

It is unclear at this point why the protein fusion construct had a smaller effect than the operon construct. Transcript analysis of *ispA* and *CnVS* in the two strains showed similar expression levels (Fig. S3 B); *ispA* was expressed slightly higher in the operon construct. It is therefore unlikely that different transcript levels play a role in metabolic output, although this might be a hint that it could be beneficial to find the correct balance of expression between all enzymes involved - higher levels of *ispA* lead to higher conversion of IPP and DMAPP toward FPP. The most likely reason for the poorer performance of the fusion protein is therefore a loss in efficiency due to impeded enzyme function or misfolding of the protein. Since other studies showed great promise in this area of research (Daletos and Stephanopoulos, 2020; Wang et al., 2021), it might therefore be interesting to further investigate different protein fusion constructs, for example by switching the order of the enzymes, as well as exploring different protein linkers. While the use of *ispA* in combination with *CnVS* was briefly described earlier (Matsudaira et al., 2020), we show that this combination of genetic components is even more productive in combination with metabolic engineering of the native pathways in *Synechocystis*, yielding improved levels of valencene. Strikingly, additional *crtE* repression of *ispA*-expressing strains with aTc further increased valencene titer up to 17.6 mg/L and 12.5 mg/L valencene. In contrast, the strains producing more valencene also show a noticeable reduction in carotenoid content, indicating that the pool of the precursors IPP and DMAPP, which are normally diverted towards carotenoid production are now available and successfully used as a substrate by CnVS. This is also consistent with earlier works, in which a common carotenoid precursor was diverted towards production of manoyl oxide (Englund et al., 2015). Surprisingly, despite the reduced pigment content coupled with the metabolic burden of valencene production, the aTc-induced cells grew remarkably well, reaching an OD_750_ of ~2.5 compared to uninduced cells, which reached an OD_750_ of ~3 after 48 h. It is possible that aTc-mediated *crtE*-repression is, in fact, transient due to the light-sensitive properties of aTc, and that after an initial rerouting of the precursor pool towards valencene, the cell returns back to its initial balanced state. While *crtE* was expected to be an essential gene due to carotenoids being an essential part of light harvesting and photoprotection, it remains unclear at this point whether the effect is transient. Nevertheless, the decrease in carotenoid levels clearly shows the expected metabolic effect. It is therefore likely that the introduced genetic alterations function as hypothesized and that a majority of the terpenoid precursor pool is in fact diverted towards valencene production. However, the molar increase in valencene corresponds to roughly three times the amount of FPP that would be made available by the reduction of carotenoids alone. Since the phytol tail of chlorophyll is also derived from GGPP, this is likely partially responsible for the discrepancy. Furthermore, metabolic feedback regulation probably plays an important role, both within the isoprenoid biosynthetic pathway and the carotenoid pathway (Cazzonelli and Pogson, 2010). Since carotenoids are heavily involved in the response to light stress (Llewellyn et al., 2020; Steiger et al., 1999), reduced carotenoid content could lead to the accumulation of ROS, thereby possibly triggering increased flux towards GGPP.

It would be highly interesting to investigate valencene production over time in order to assess whether the generated strain produces stable metabolic output over a longer amount of time, or whether the cell returns to its pigmented state. We therefore observed the behavior of the best-performing strain, ΔΔ *crtE*↓ IspA:CnVS-op + aTc, over five days. Three replicates were precultured in 30 mL BG11 in non-baffled flasks, induced with 5 mM L-rhamnose and 10 ng/mL aTc, overlaid with 3 mL dodecane, and observed over five days. Fig. S5 shows the volumetric daily production rates of the strain, as well as total valencene accumulation and OD_750_. While the cell density reaches a plateau after four days, valencene is continuously produced. There is a strong depletion of pigments in the production strain (Fig. S5B), both in carotenoid and chlorophyll content. Despite this strong phenotype, the cells appear to retain some level of productivity. However, the pigmentation, as well as the growth halt further indicates that the strain can be further optimized to regain some productivity likely lost due to the loss of photosynthetic efficiency.

The individual yields of each strain in terms of culture volume, dry cell weight (DCW), and cell density are summarized in Table 1.

**Table 1:** Individual valencene production performance of strains investigated in this work. Downward arrow represents CRISPRi-mediated repression. ΔΔ represents the Δshc, Δsqs double mutant. All values shown represent the mean ± the standard deviation of three biological replicates.

Strain	Genotype	Genes expressed from plasmid	Yield [mg/L]	Yield [mg/gDCW]	Yield [mg/OD_750_]
WT	Non-motile wild type Synechocystis sp. PCC 6803	–	n.d.	n.d.	n.d.
Δshc,Δsqs	Δshc,Δsqs	–	n.d.	n.d.	n.d.
ΔΔcrtE↓	Δshc,Δsqs,ΔpsbA1::crtE↓	–	n.d.	n.d.	n.d.
WT CnVS	–	CnVS	3.2 ± 0.25	4.5 ± 0.43	1.7 ± 0.13
ΔΔ CnVS	Δshc,Δsqs	CnVS	4.7 ± 0.06	6.4 ± 0.52	2.4 ± 0.06
ΔΔcrtE↓CnVS-aTc	Δshc,Δsqs,crtE↓	CnVS	3.6 ± 0.47	3.7 ± 0.42	1.5 ± 0.18
ΔΔcrtE↓CnVS + aTc	Δshc,Δsqs,crtE↓	CnVS	2.0 ± 0.12	2.3 ± 0.18	0.9 ± 0.06
ΔΔcrtE↓CnVS-op-aTc	Δshc,Δsqs,crtE↓	ispA, CnVS(operon)	12.5 ± 0.44	9.8 ± 0.54	3.9 ± 0.12
ΔΔcrtE↓CnVS-op + aTc	Δshc,Δsqs,crtE↓	ispA, CnVS(operon)	17.6 ± 0.71	19.0 ± 0.62	7.1 ± 0.14
ΔΔcrtE↓CnVS-fus-aTc	Δshc,Δsqs,crtE↓	ispA, CnVS(fusion)	6.0 ± 0.27	4.9 ± 0.25	1.9 ± 0.08
ΔΔcrtE↓CnVS-fus + aTc	Δshc,Δsqs,crtE↓	ispA, CnVS(fusion)	12.5 ± 2.15	12.6 ± 2.18	5.0 ± 0.84

## 4 Conclusion & outlook

For the redirection of metabolic flux towards the heterologous production of terpenoids, in this case the sesquiterpene valencene, we identified the native carotenoid pool of *Synechocystis* as a major target. We were able to demonstrate the capability of *Synechocystis* to divert terpene precursors by I. Deletion of native metabolic pathways not essential to the central metabolism, markerless Δ*shc* and Δ*sqs*, II. Conditional gene repression of a major component in the terpenoid pathway, *crtE*, and III. Introduction of heterologous enzymes, *ispA* and CnVS, with functions tailored to the specific production of our target molecule. With these strategies, we were able to successfully overcome some of the native pathway bottlenecks in cyanobacteria, while simultaneously exploiting their native ability of producing terpene compounds. Observing the best-producing strain over time also showed that there is even more potential towards optimization towards a more robust production strain. We believe that this delicate balance between cell viability in terms of conversion of light to energy, but also protection from light stress on the one hand, and improved productivity is an important step towards utilizing these photosynthetic organisms in a more continuous, industrial-scale application. Future studies of long-term effects of metabolic engineering of strains will certainly help improve engineering strategies towards industrially relevant utilization of cyanobacterial chassis.

Furthermore, valencene also serves as an intermediate, which can be converted to nootkatone via cytochrome P450 enzymes (CYPs). CYPs that function as monooxygenases are found in many plant species, often membrane-bound and dependent on the availability of oxygen and NADPH. In terms of functionality, cyanobacteria might be especially suitable for the application of engineered CYPs: They already contain endogenous cytochromes P450, for which oxygen and NADPH are readily available via photosynthetic activity, while this can be a limiting factor in heterotrophs.

## Funding

This project was financially supported by the CLIB-Competence Centre Biotechnology (CKB) funded by the European Regional Development Fund ERDF (34.EFRE-0300096).

## Declaration of competing interest

The authors declare that they have no known competing financial interests or personal relationships that could have appeared to influence the work reported in this paper.

## Author statement

Maximilian Dietsch: Conceptualization, Investigation, Methodology, Writing - Original Draft, Writing - Review & Editing, Visualization, Data Curation. Anna Behle: Conceptualization, Investigation, Writing - Original Draft, Writing - Review & Editing, Visualization, Data Curation. Philipp Westhoff: Methodology, Data Curation. Ilka M. Axmann: Supervision, Writing- Reviewing and Editing.
