# Supplementary material for: Metabolic engineering of Synechocystis sp. PCC 6803 for the photoproduction of the sesquiterpene valencene
Source: Metab Eng Commun. 2021 Aug 13;13:e00178. doi: 10.1016/j.mec.2021.e00178 (PMC8382996; doi:10.1016/j.mec.2021.e00178)
Supplement: Multimedia component 1 [file mmc1.docx]

Metabolic engineering of *Synechocystis* sp. PCC 6803 for the photoproduction of the sesquiterpene valencene

Maximilian Dietsch^1*^, Anna Behle^1*^, Philipp Westhoff^2^, Ilka M. Axmann^1^

^1^Institute for Synthetic Microbiology, Department of Biology, Heinrich Heine University Düsseldorf, Düsseldorf, Germany.

^2^Plant Metabolism and Metabolomics Laboratory, Cluster of Excellence on Plant Sciences (CEPLAS), Heinrich Heine University Düsseldorf, D-40001 Düsseldorf, Germany

*These authors contributed equally to this work

# Supporting Information

Fig. S1: Supplementary information on the markerless mutants ∆*shc* and ∆*shc*, ∆*sqs*.

Fig. S2: Pigment quantification and growth behavior of the *crtE* knock-down strain.

Fig. S3: Western Blot and qRT-PCR analysis of IspA:CnVS fusion vs. operon strains.

Fig. S4: Mass spectra comparison of samples with reference

Fig. S5: Physiological changes and valencene production in ∆∆ *crtE*↓ IspA:CnVS-op +aTc*.*

Fig. S6: Quantification of possible valencene loss via evaporation or degradation

Supplementary Table S1: Cq values for *shc* and *sqs* in WT and knock-out strains.

Supplementary Table S2: Detailed descriptions and sequences of all relevant genetic modules used in this work.


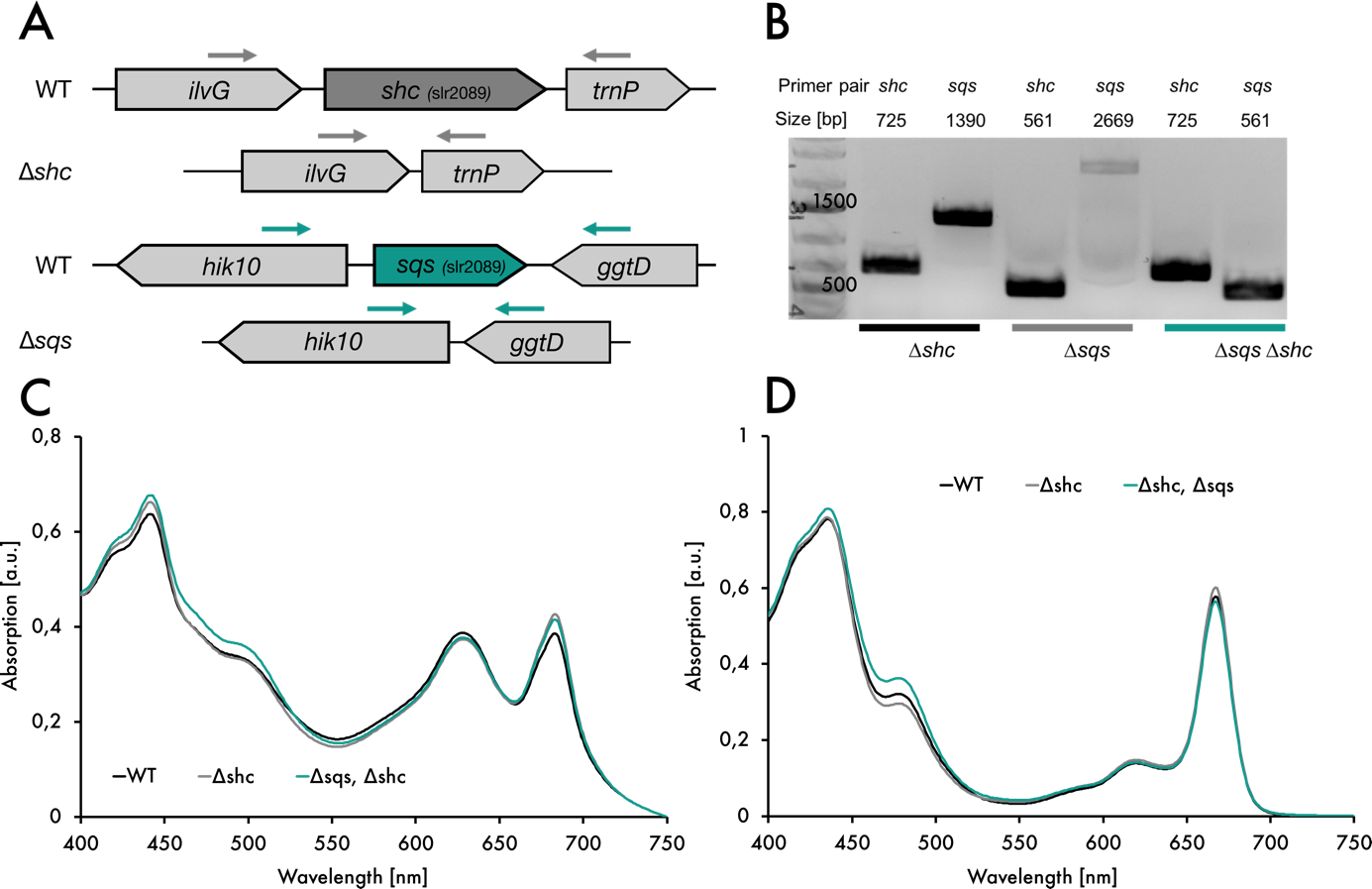


Fig. S1: Supplementary information on the markerless mutants ∆*shc* and ∆*shc*, ∆*sqs*.
A: Schematic overview of markerless mutant genotypes. Arrows denote primers used for colony PCR.

B: PCR analysis of single and double mutant strains using oligonucleotides that bind outside of the affected area. Primer pair and expected sizes are shown above. Thermo 1kb+ ladder was used as size standard. C: Whole cell spectra of WT, ∆*shc* and double mutant. Spectra were baseline-corrected by subtracting the absorption at 750 nm. D: Spectra of methanol-extracted cells from WT, ∆*shc* and double mutant strains.


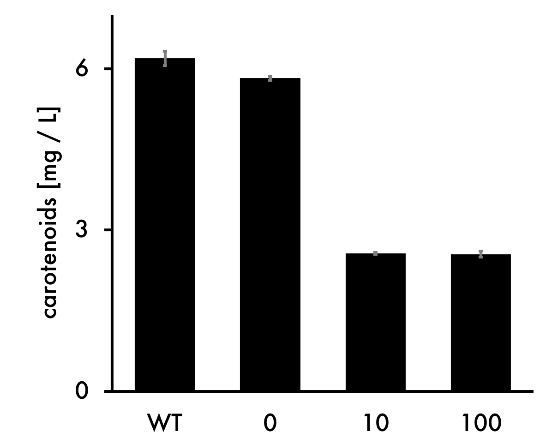

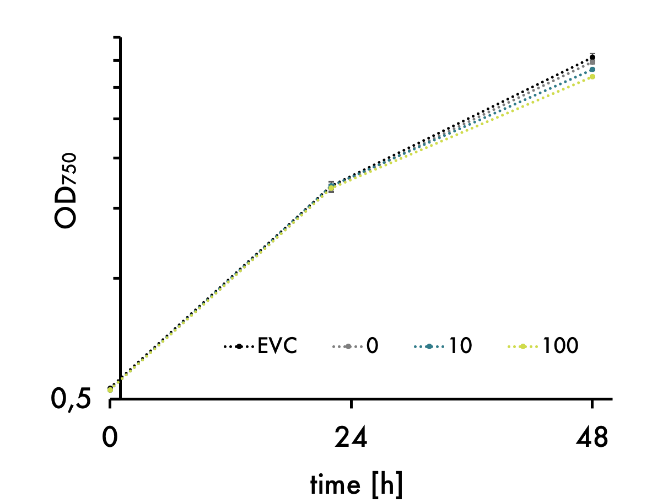


B

A

Fig. S2: Pigment quantification and growth behavior of the *crtE* knock-down strain.
A: Carotenoid content of ∆*shc*, ∆*sqs* mutant expressing dCas9 only (WT) compared to ∆*shc*, ∆*sqs* mutant expressing both dCas9 and the *crtE* sgRNA, induced with 0, 10, and 100 ng/mL aTc. Carotenoids were quantified as described in Material & Methods, section 2.5. B: Growth behavior of aforementioned strains.


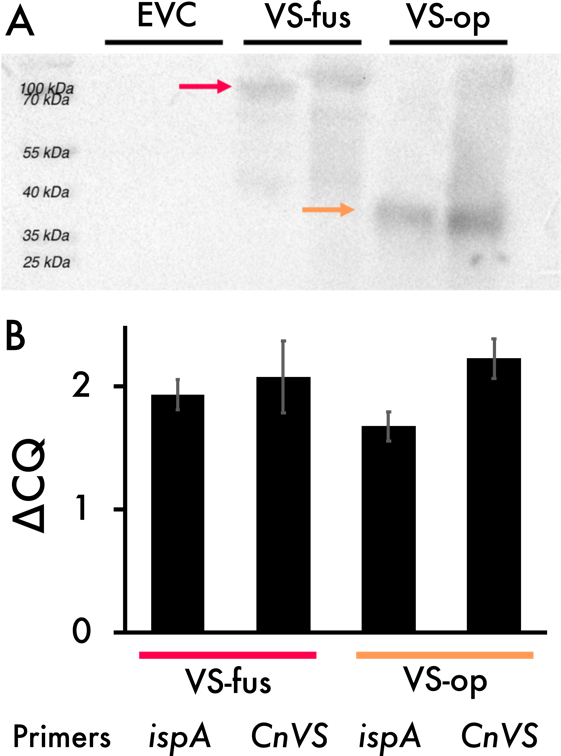


Fig. S3: Western Blot and qRT-PCR analysis of IspA:CnVS fusion vs. operon strains.
A: Western Blot analysis of IspA:CnVS protein fusion (VS-fus) and IspA:CnVS operon (VS-op). The fusion protein N-FLAG-IspA-CnVS corresponds to a size of ~105 kDa, while N-FLAG-IspA in the operon construct corresponds to about ~35 kDa. B: ∆CQ values of qRT-PCR performed on both strains using either ispA or CnVS primers, as denoted below. ∆CQ values were calculated by subtracting the CQ value of the housekeeping gene rnpB from each CQ value. A higher ∆CQ value corresponds to a lower transcript amount.

#

Fig. S4: Mass spectra comparison of samples with reference.
Top: Mass spectra of wild type expressing CnVS. Bottom: Reference mass spectra of (+)-valencene. Middle: Direct comparison of top and bottom spectra.

Table S1: Cq values for *shc* and *sqs* in WT and knock-out strains.

Values represent the mean and standard deviation of three biological replicates. Cq values were obtained via qRT-PCR. Sample values above 30 were defined as not containing any template.

| Strain | Target gene | Cq value |
| --- | --- | --- |
| Wild type | *sqs* | 21.2 ± 0.3 |
|  | *shc* | 21.8 ± 0.2 |
| ∆*shc* | *sqs* | 21.1 ± 0.2 |
|  | *shc* | 33.4 ± 0.6 |
| ∆*shc*; ∆*sqs* | *sqs* | 31.5 ± 0.8 |
|  | *shc* | 35.5 ± 1.2 |
| No template control | *sqs* | 34.4 |
|  | *shc* | n. def. |

**
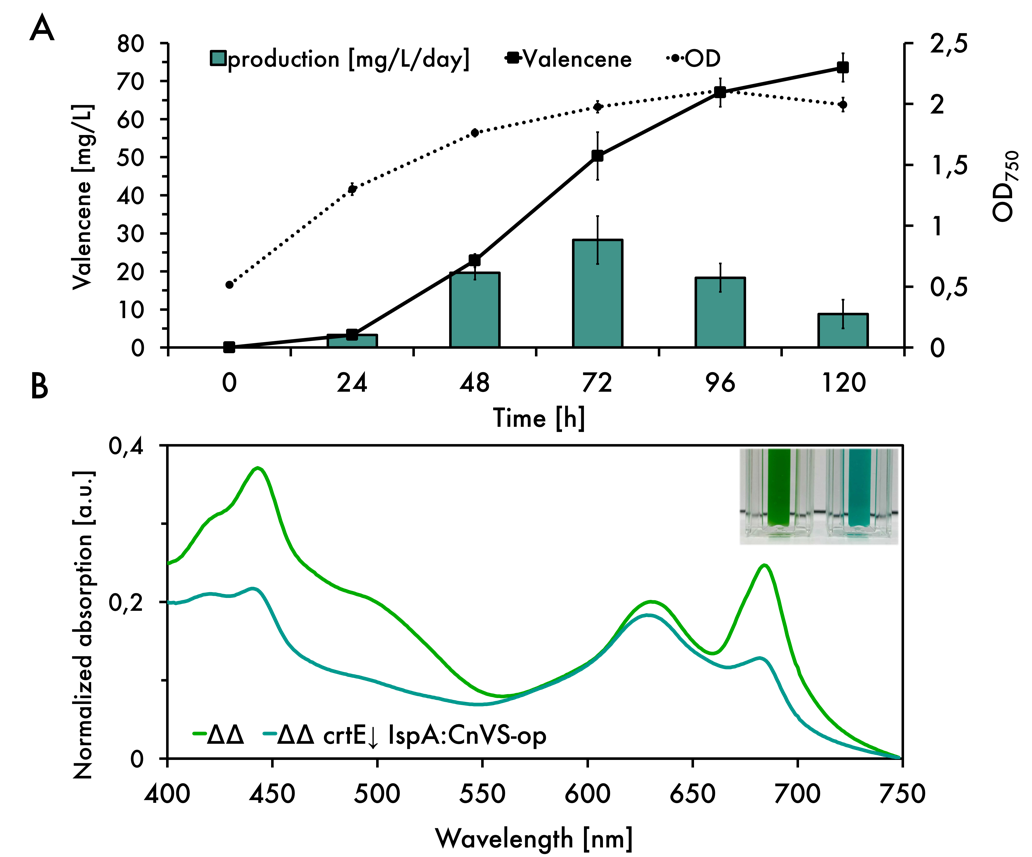
**

Fig. S5: Physiological changes and valencene production in ∆∆ crtE↓ IspA:CnVS-op +aTc. The strain was cultured in biological triplicates over five days in shake flasks overlaid with 10% dodecane. The dodecane layer, as well as the culture, were sampled daily for valencene quantification and cell density (OD_750_), respectively. A: Volumetric accumulation of valencene (square symbols, continuous line) and cell density (round symbols, dotted line). The volumetric production per day is shown as blue bars, corrected for the sample removed each day. B: Whole cell spectra of double mutant (green) and ∆∆ crtE↓ IspA:CnVS-op +aTc (blue) after 120 h cultivation. Spectra were baseline-corrected by subtracting the absorption at 750 nm. An image of the cuvettes is embedded for better visualization of the color difference (left: double mutant, right: ∆∆ crtE↓ IspA:CnVS-op +aTc).

**
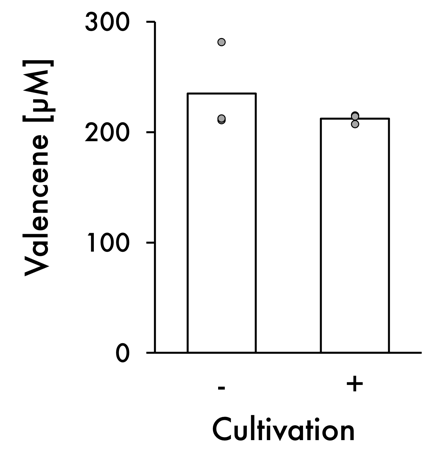
**

Fig. S6: Quantification of possible valencene loss via evaporation or degradation. A dodecane sample containing 225 µM valencene was used to overlay a Synechocystis wild type culture, which was grown for 48 h in technical triplicates. Both the initial sample (-) and the sample recovered from the culture (+) was measured and compared. The bars represent the mean of the three technical replicates, each of which is shown in grey. No significant difference was observed (t-test, P=0.39).

**Table S2: Detailed descriptions and sequences of all relevant genetic modules used in this work.**

| **Name** | **Part type** | **Sequence** | **Origin** | **Notes** |
| --- | --- | --- | --- | --- |
| P_rha_ | Promoter | gccacaattcagcaaattgtgaacatcatcacgttcatctttccctggttgccaatggcccattttcctgtcagtaacgagaaggtcgcgaattcaggcgctttttagactggtcgtaatgaa | (Behle *et al*., 2020) |  |
| RBS* | RBS | tactagagtagtggaggttactag | (Behle *et al*., 2020) |  |
| P_J23119_ | Promoter | ttgacagctagctcagtcctaggtataatgctagc | (Behle *et al*., 2020) | Bba_J23119 in iGEM registry |
| Bba_0034 | RBS | aaagaggagaaatactag | (Behle *et al*., 2020) | Bba_B0034 in iGEM registry |
| rhaS | CDS | atgaccgtattacatagtgtggatttttttccgtctggtaacgcgtccgtggcgatagaaccccggctcccgcaggcggattttcctgaacatcatcatgattttcatgaaattgtgattgtcgaacatggcacgggtattcatgtgtttaatgggcagccctataccatcaccggtggcacggtctgtttcgtacgcgatcatgatcggcatctgtatgaacataccgataatctgtgtctgaccaatgtgctgtatcgctcgccggatcgatttcagtttctcgccgggctgaatcagttgctgccacaagagctggatgggcagtatccgtctcactggcgcgttaaccacagcgtattgcagcaggtgcgacagctggttgcacagatggaacagcaggaaggggaaaatgatttaccctcgaccgccagtcgcgagatcttgtttatgcaattactgctcttgctgcgtaaaagcagtttgcaggagaacctggaaaacagcgcatcacgtctcaacttgcttctggcctggctggaggaccattttgccgatgaggtgaattgggatgccgtggcggatcaattttctctttcactgcgtacgctacatcggcagcttaagcagcaaacgggactgacgcctcagcgatacctgaaccgcctgcgactgatgaaagcccgacatctgctacgccacagcgaggccagcgttactgacatcgcctatcgctgtggattcagcgacagtaaccacttttcgacgctttttcgccgagagtttaactggtcaccgcgtgatattcgccagggacgggatggctttctgcaataa | *E. coli* | Arg214Leu mutation |
| natR | CDS | ctaggggcagggcatgctcatgtagagcgcctgccgctcgccgtccgaggcggtgccgtcgtacagggcggtgtccaggccgcagagggtgaaccccatccgccggtacgcgtggatcgccggtgcgttgacgttggtgacctccagccagaggtgcccggcgccccgctcgccggcgaactccgtcgcgagccccatcaacgcgcgcccgaccccgtgcccccggtgctccggggcgacctcgatgtcctcgacggtcagccggcggttccacgccgagtacgagatgaccacgaagcccgccaggtcgccgtcgtccccgtacgcgacgaacgtccgggagtccgggtcgccgtcctccccgtcgtccgattcgtcgtccgattcgtcgtcggggaacaccttggtcaggggcgggtccaccggcacctcccgcagggtgaagccgtccccggtggcggtgacgcggaagacggtgtcggtggtgaaggacccatccagtgcctcgatggcctcggcgtcccccgggacactggtgcggtagcggtaagccgtgtcgtcaagagtggtcat | *Streptomyces* |  |
| N-FLAG | N-terminal FLAG tag | ATGGCTAGCgattataaagatcatgatggcgattataaagatcatgatattgattataaagatgatgatgataaaGGT | (Wiegard *et al*., 2013) | Contains an NheI restriction site after the start codon; and a single glycin linker at the end (ggt) |
| ispA | CDS | atggacttcccgcagcagctagaggcctgtgtgaagcaagcaaatcaagcgctcagccgttttatcgccccactacccttccaaaacactcctgtcgtggaaactatgcaatacggcgcgttactgggaggcaaacgattgcggccgtttttggtctatgccaccggccacatgttcggagtgagcaccaacacactcgacgctccagctgcggcagttgagtgtattcacgcatattctttgatccatgatgatctacccgcaatggatgacgatgacctgcgtcgaggcttacctacttgtcacgtcaagttcggcgaagcgaatgcaattctagcgggcgacgcgttgcaaactttggctttctccattttatccgatgccgatatgccggaagtctctgaccgcgaccgaatttccatgatctctgaactcgccagcgcgagcggaatcgccggaatgtgcggtggtcaagccctggacttggatgcggaaggaaaacacgttcctctcgatgcgttagaacgaatccatcgtcataagactggtgctctcatccgtgccgcagttcgactcggggcgttgagcgctggtgacaagggacgacgcgctttacctgtgctcgacaaatacgctgagtctattggtctcgcattccaagttcaggacgacattctcgacgttgtgggagacacggccacgctggggaaacggcagggtgcggaccagcagctcgggaaaagcacgtacccggctttactcggtctcgaacaagcacgcaagaaggcgcgggacttgattgatgatgctcgccagtccctgaagcagttagcagaacagtctctcgacacttccgctttggaggctctcgcggactatattattcagcggaataaataa | *E. coli* | Gene was codon optimized for *Synechocystis* sp. PCC 6803 |
| CnVS | CDS | atggcggaaatgtttaatgggaacagcagtaatgacgggtcctcttgtatgcctgtgaaggacgctctccgtcggactggaaaccatcatccaaacttatggacggatgactttatccaaagcctgaatagtccatattctgactctagttatcacaaacatcgagaaattctaatcgacgaaatccgcgacatgtttagcaatggtgagggtgatgaatttggcgtgttggaaaacatctggtttgtggatgtagtacagcggctcgggattgaccgtcatttccaggaagagatcaaaacagcactggattacatctataaattttggaaccacgattctatttttggggatctgaacatggtcgctctcggttttcgtattttgcggctaaatcgttacgtagcgagtagcgatgtgttcaaaaaattcaagggtgaggagggccaattttctggcttcgagtccagtgaccaagacgccaagttggaaatgatgttaaatttgtataaggcatccgaattggatttcccagacgaggatattctaaaggaggcccgcgcgttcgcaagtatgtacctcaagcacgttatcaaggaatatggggacatccaagaaagcaagaacccactcctaatggaaattgaatacacttttaagtatccgtggcgctgtcgcttaccacgtttagaggcgtggaacttcatccatattatgcgccaacaggactgtaatattagcctggcgaacaacctatacaagattccaaaaatttacatgaaaaaaattctggagttggccattctggatttcaacattctacagagtcagcatcaacacgagatgaagctcatctctacatggtggaaaaactcttccgctattcaactcgatttttttcgccatcgacatatcgagagctacttttggtgggcctcccccctatttgaacctgagttctccacctgtcgcattaactgcacgaagctgagcactaagatgtttctattggacgacatctatgatacgtatggtacggtcgaggaattaaagccttttacgacaactctaactcgatgggatgtatccacggttgataatcatccggattatatgaaaattgcgttcaatttttcctacgaaatctataaagagattgcatccgaagccgagcgtaagcacgggccttttgtttacaaatatctgcaaagttgttggaagagttacattgaagcatatatgcaggaagcggagtggattgcttccaaccacattcctggattcgatgaatacctcatgaacggtgtcaagtcctccggtatgcgtattttgatgattcatgctctcatcctcatggatacgccattgagcgatgaaatcttagaacagctggacattccaagttctaaaagtcaggcgctcttgagcctaatcacacggctagtcgatgacgtcaaggatttcgaagatgagcaggcccacggagagatggcaagtagtatcgagtgttatatgaaagataaccacggttccacacgggaggatgcgttaaactacctcaagatccggatcgagagctgtgttcaggaactaaataaagaactgctagaaccaagcaacatgcacggcagtttccggaacttgtatttgaacgtaggaatgcgtgtcatcttcttcatgctcaacgacggtgatttgtttacgcattccaaccgtaaagaaatccaagacgcaatcactaaattttttgtcgaacccattatcccgaagctctaa | *Callitropsis nootkatensis* | Gene was codon optimized for *Synechocystis* sp. PCC 6803 |
| GGGGS | protein linker | ggcggtggcggatcc | (Hu *et al*., 2017) |  |
| sgRNA crtE | sgRNA | AAGTCGGTTCGTGTTTGTT |  |  |
|  | total sgRNA part | tccctatcagtgatagagattgacatccctatcagtgatagagatactgggagctaaagtcggttcgtgtttgttgttttagagctagaaatagcaagttaaaataaggctagtccgttatcaacttgaaaaagtggcaccgagtcggtgcttttttt | (Yao *et al*., 2020) | P_L22_, sgRNA, dCas9-binding |
| T7-term | Terminator | ggctcaccttcgggtgggcctttctgcg |  |  |

Supplementary References

Behle, Anna, Pia Saake, Anna T. Germann, Dennis Dienst, und Ilka M. Axmann. „Comparative Dose–Response Analysis of Inducible Promoters in Cyanobacteria“. *ACS Synthetic Biology* 9, Nr. 4 (17. April 2020): 843–55. https://doi.org/10.1021/acssynbio.9b00505.

Hu, Yating, Yongjin J. Zhou, Jichen Bao, Luqi Huang, Jens Nielsen, und Anastasia Krivoruchko. „Metabolic Engineering of *Saccharomyces* *Cerevisiae* for Production of Germacrene A, a Precursor of Beta-Elemene“. *Journal of Industrial Microbiology & Biotechnology* 44, Nr. 7 (Juli 2017): 1065–72. https://doi.org/10.1007/s10295-017-1934-z.

Wiegard, Anika, Anja K. Dörrich, Hans-Tobias Deinzer, Christian Beck, Annegret Wilde, Julia Holtzendorff, und Ilka M. Axmann. „Biochemical Analysis of Three Putative KaiC Clock Proteins from *Synechocystis* Sp. PCC 6803 Suggests Their Functional Divergence“. *Microbiology* 159, Nr. Pt_5 (1. Mai 2013): 948–58. https://doi.org/10.1099/mic.0.065425-0.

Yao, Lun, Kiyan Shabestary, Sara M. Björk, Johannes Asplund-Samuelsson, Haakan N. Joensson, Michael Jahn, und Elton P. Hudson. „Pooled CRISPRi Screening of the Cyanobacterium *Synechocystis* Sp PCC 6803 for Enhanced Industrial Phenotypes“. *Nature Communications* 11, Nr. 1 (Dezember 2020): 1666. https://doi.org/10.1038/s41467-020-15491-7.
